# A Mathematical Modeling Approach to Uncover Factors Influencing the Spread of *Campylobacter* in a Flock of Broiler-Breeder Chickens

**DOI:** 10.3389/fmicb.2020.576646

**Published:** 2020-10-28

**Authors:** Thomas Rawson, Robert Stephen Paton, Frances M. Colles, Martin C. J. Maiden, Marian Stamp Dawkins, Michael B. Bonsall

**Affiliations:** ^1^Mathematical Ecology Research Group, Department of Zoology, University of Oxford, Oxford, United Kingdom; ^2^Peter Medawar Building for Pathogen Research, Department of Zoology, University of Oxford, Oxford, United Kingdom; ^3^National Institute for Health Research, Health Protection Research Unit in Gastrointestinal Infections, University of Oxford, Oxford, United Kingdom; ^4^Department of Zoology, John Krebs Field Station, University of Oxford, Oxford, United Kingdom

**Keywords:** *Campylobacter*, mathematical model, Bayesian model, poultry, transmission dynamics

## Abstract

Despite continued efforts to improve biosecurity protocols, *Campylobacter* continues to be detected in the majority of commercial chicken flocks across Europe. Using an extensive data set of *Campylobacter* prevalence within a chicken breeder flock for over a year, multiple Bayesian models are presented to explore the dynamics of the spread of *Campylobacter* in response to seasonal variation, species-specificity, bird health, and total colonization prevalence. These models indicated that birds within the flock varied greatly in their response to bacterial challenge, and that this phenomenon had a large impact on the overall prevalence of different species of *Campylobacter*. *Campylobacter jejuni* appeared more frequently in the summer, while *Campylobacter coli* persisted for a longer duration, amplified by the most susceptible birds in the flock. Our study suggests that strains of *Campylobacter* that appear most frequently likely possess no demographic advantage, but are instead amplified due to the health of the birds that ingest it.

## Introduction

Poultry meat has been decisively implicated as a leading infection route for campylobacteriosis in humans (EFSA Panel on Biological Hazards, 2011). With an estimated 450,000 cases a year in the UK, ~10% of which result in hospitalization (Strachan and Forbes, 2010), *Campylobacter* presents an important public health challenge, and an estimated £50 million annual economic burden to the UK (Tam and O'Brien, 2016). An investigation by Public Health England indicated the extent to which *Campylobacter* spp. dominated the commercial poultry industry: seventy-three percent of supermarket chicken carcasses were found to contain *Campylobacter* and seven percent of the outer packaging was similarly contaminated (Jorgensen et al., 2015). Consequently, preventing the spread of the bacteria to humans by reducing the number of colonized broiler flocks, i.e., chickens grown specifically for meat, at slaughter is as an urgent endeavor (Wilson et al., 2008).

Current attempts at tackling outbreaks of *Campylobacter* have largely focused around on-farm biosecurity measures; however, little impact in reducing incidence has been demonstrated (Hermans et al., 2011). This is predominantly due to the aggressive rate of proliferation once *Campylobacter* has entered a flock, and further complicated by uncertainty in the exact route of primary colonization. Specifically designed prevention methods are also marred by high genetic diversity of *Campylobacter* spp. (Tresse et al., 2017).

*Campylobacter* has historically been considered to be a gastrointestinal commensal of chickens, but recent studies suggest that, at least in some circumstances, they are pathogenic (Humphrey et al., 2014; Wigley, 2015). We here refer to birds being “colonized” and *Campylobacter* spp. “shed” where they could be detected from samples. Once an initial bird has become colonized by *Campylobacter*, colonization of the rest of the flock occurs very rapidly, usually within the course of a week (Evans and Sayers, 2000; Shreeve et al., 2000; Stern et al., 2001). The bacteria are spread via the fecal-oral route. After becoming newly-colonized, the host broiler spends a brief period in a non-infectious incubation period, before excreting the bacteria in its fecal and caecal matter. Surrounding susceptible broilers are then exposed to this via coprophagy (Shanker et al., 1990).

Understanding of the spread of *Campylobacter* is hindered by incomplete understanding of the transmission dynamics of the bacteria at farm level. Multiple strains of *Campylobacter* can simultaneously inhabit broiler flocks (Höök et al., 2005), with some strains appearing to dominate the flock at different times (De Cesare et al., 2008; Kudirkienė et al., 2010). It has been suggested that these dynamical behaviors are driven by the appearance of demographically superior strains that outcompete other strains (Calderón-Gómez et al., 2009) within the gut. However, another study suggests that strains are lost or transmitted randomly, regardless of their genotypic differences (Grant et al., 2005). Indeed, recent mathematical modeling approaches have demonstrated that stochastic simulations can effectively capture the broad dynamical differences between strains of equal demographic ability (Rawson et al., 2019).

An area of more recent study is the role played by “super shedders,” birds who consistently shed high amounts of *Campylobacter* in their feces, in the transmission dynamics of *Campylobacter* within a flock. The impact of “super shedders” has been well-documented as a key factor in the rapid spread of *Salmonella* throughout chicken flocks (Gopinath et al., 2012; Menanteau et al., 2018), but the impact on the dynamics of *Campylobacter* spread within broiler flocks is not well-studied. These “super-shedders” have been found experimentally to have fewer circulating heterophilic cells, but this does not appear to be a genetically acquired trait, nor the result of differences in adaptive immunity (Barrow et al., 2004). The presence of such super shedders in broiler flocks has been observed in an experimental study measuring *Campylobacter* prevalence (Achen et al., 1998), and it is reasonable to assume that this could have implications for the transmission dynamics within a flock. Despite the lack of studies amongst chickens, variation in fecal shedding of Campylobacter has been detected in cattle (Rapp et al., 2012).

Some factors affecting transmission are well-reported, if incompletely understood. The effect of seasonal variation on both the carriage rate, and number of *Campylobacter* found in the caeca, of colonized chickens has been noted (Wallace et al., 1997), with an increase often observed in the spring or summer. The exact timing of these peaks, however, varies within and among countries (Kovats et al., 2005), and experimental work is not always able to detect such an effect (Humphery et al., 1993). Less well-investigated is the impact of different species of *Campylobacter* competing within a flock. *C. jejuni*, the most common species, has been found in ~90% of British chicken flocks, compared with *C. coli* appearing in 10% of flocks (Jorgensen et al., 2011). This ratio has been reported by other studies in broiler flocks (Bull et al., 2006), with species rarely both being simultaneously present. It is not understood whether this is due to established strains suppressing new strains from emerging, demographic differences, or the short-lifespan of commercial broiler flocks not providing enough time for multiple species to colonize a flock. Under laboratory conditions, *C. coli* has been shown to have lower growth rates, motility, and invasiveness than *C. jejuni* (Aroori et al., 2013), potentially explaining its rarer appearance in chicken flocks. There is also some suggestion that *C. coli* is more commonly isolated from older, free-range, birds (Colles et al., 2008).

This study explores the impact of multiple factors on the transmission of multiple sequence types (STs) of *Campylobacter* amongst individual birds within a flock across 51 weeks. A broiler breeder flock, i.e., the parents of broiler/meat birds, was studied rather than a broiler flock, since the production period of around a year, compared to a small number of weeks for broiler birds, allows much greater potential to study the interaction of different *Campylobacter* strains over time. When interpreting the results, however, it should be noted that broiler breeder flocks will differ from broiler flocks with respect to, for example, host genetics, age, feed, and flock density. The majority of the *Campylobacter* genotypes (STs), however, have been isolated from other chicken flocks, most typically housed commercial broiler flocks (Colles et al., 2015).

We use a robust data set from Colles et al. (2015), which is currently the best available for monitoring the *Campylobacter* strain dynamics amongst individual birds within a commercially reared flock across 51 weeks. Through a Bayesian modeling approach we show the range of receptiveness to colonization throughout the flock, and highlight the role that more-susceptible, “super-shedder,” birds play in driving disease. The impact of seasonal variation is also investigated, and specific attention is given to differences between species of *Campylobacter*, so as to understand how certain strains persist at higher levels throughout the flock. Seven exploratory models are presented, each investigating a specific research question, analyzing the transition probabilities at both a flock-wide, and individual level.

A Bayesian approach is considered for this study due to the methodology's innate strengths in analyzing incomplete data (Dorazio, 2016), and enabling efficient inference of missing data. Numerical computations were carried out using the Just Another Gibbs Sampler (JAGS) program (Plummer, 2007), a Markov chain Monte Carlo (MCMC) sampling program utilizing Gibbs sampling.

## Data

The field data used for this study were originally presented in Colles et al. (2015). Within a flock of 500 broiler breeders, 200 birds were labeled with leg-rings and monitored for a total of 51 weeks. Each week, 75 unique birds were picked at random from the labeled 200, and a swab was taken of the cloacal opening. These swabs were then tested for the presence of *Campylobacter* through standard culture methods, and positive samples were then genotyped by multi-locus sequence typing (MLST) of seven house-keeping genes, enabling the sequence type (ST) and species of the *Campylobacter* isolate to be specified. Further experimental details can be found in the original publication (Colles et al., 2015).

**Figure 1:**
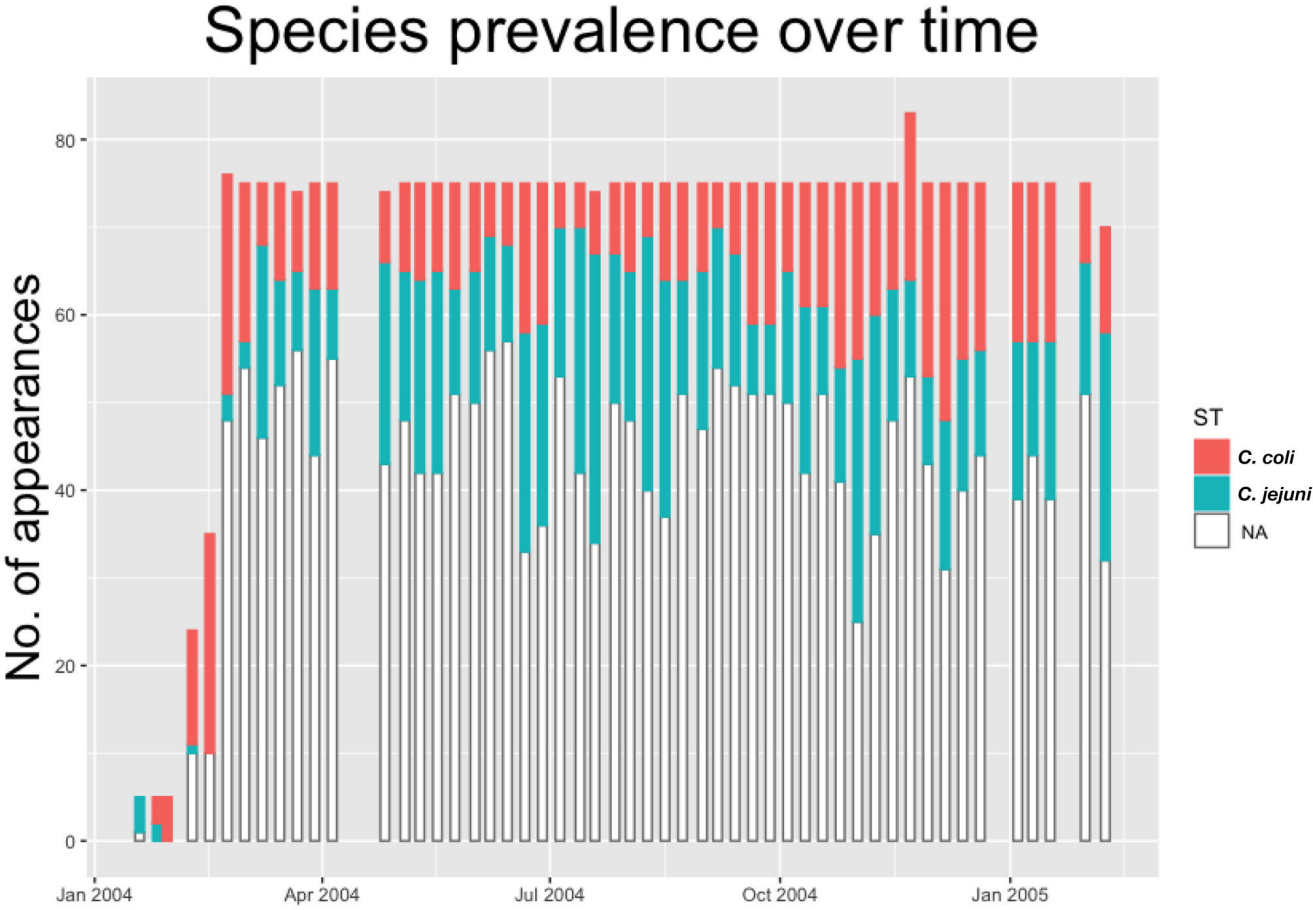
Histogram showing the count of positive and negative samples from a breeder flock for different species of *Campylobacter*. White “NA” counts represent samples that were negative for *Campylobacter*.

As such we build a dataset providing information on real-time evolution of *Campylobacter* prevalence and diversity throughout the flock. This is shown below in Figure 1, with all positive samples classified by species of *Campylobacter*.

**Figure 2:**
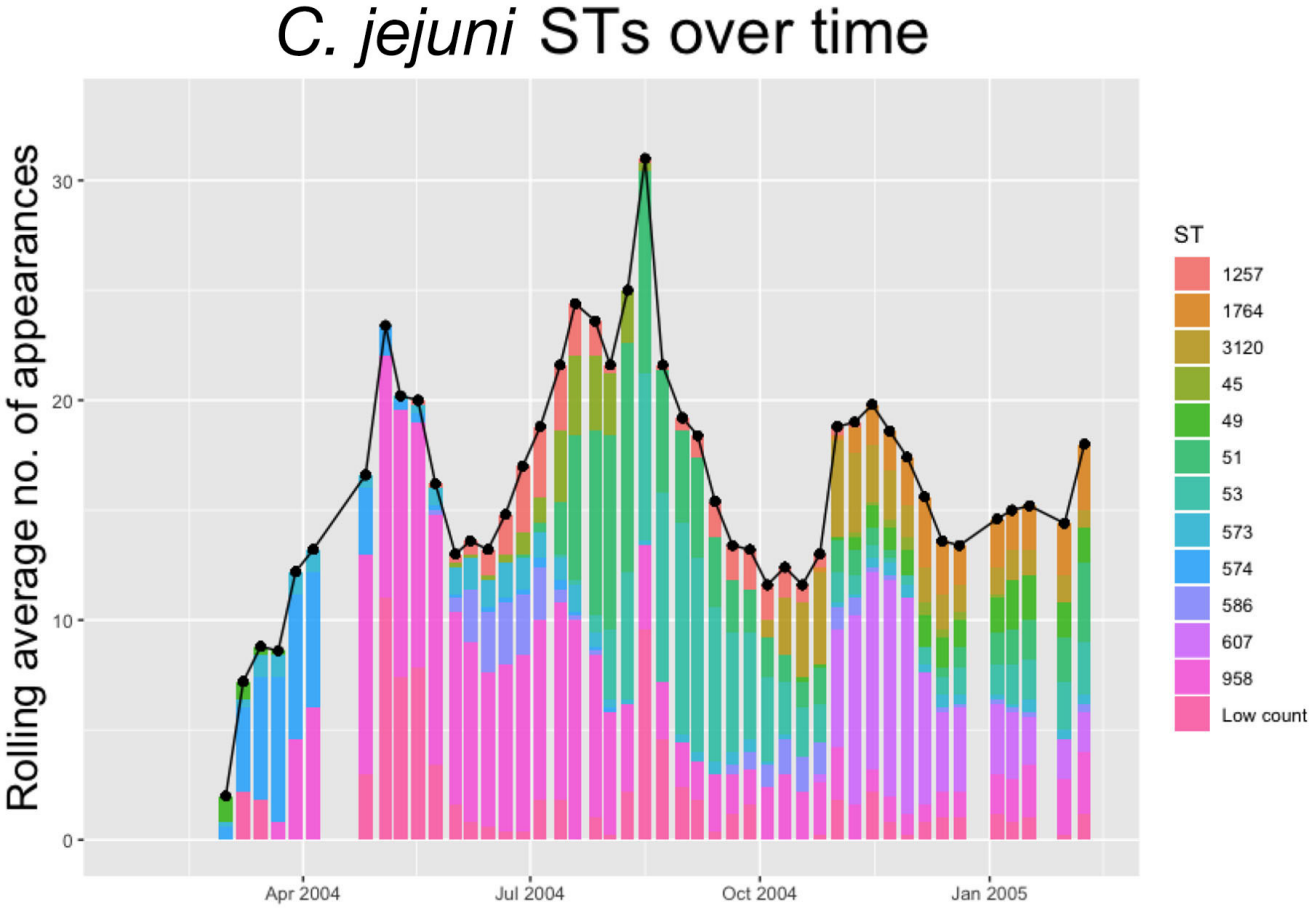
The 5-weeks rolling average number of positive samples for *Campylobacter jejuni*, with both the total number and separate ST averages. STs that appear <20 times throughout the entire experiment are amalgamated into a group “Low Count”.

**Figure 3:**
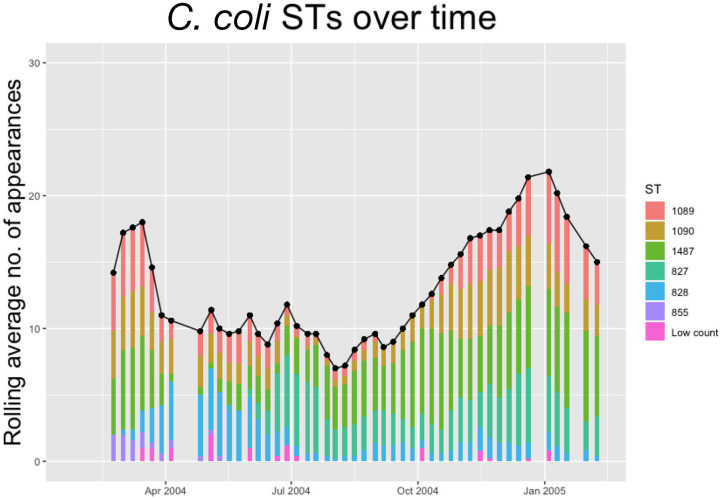
The 5-weeks rolling average number of positive samples for *Campylobacter coli*, with both the total number and separate ST averages. STs that appear <10 times throughout the entire experiment are amalgamated into a group “Low Count”.

Within each species, multiple STs are recorded. In Figures 2, 3 below we plot the 5 weeks moving averages of total positive samples for each species. Beneath each point we plot a histogram showing how this average is split between the competing STs.

We notice from Figures 2, 3 that there are more unique STs of *C. jejuni* than *C. coli*, despite both species existing at roughly equal levels. We also see that *C. jejuni* appears to peak in the summer, around the August period, coinciding with a dip in the population of *C. coli* STs. Within each species we can observe that different ST populations grow and shrink across the study period. For example, within Figure 2 we see that the summer peak is dominated by the prevalence of ST 51 and 53, however by November/December, this population shrinks, and instead ST 607 rapidly increases in population.

Figures 2, 3 effectively illustrate the key research questions tackled by this study. Namely, why do some STs seem to exist at higher quantities and persevere better than other STs which may die out? Do the dynamical behaviors of species and STs correlate to any particular trait? We investigate what mechanisms are dynamically driving these observed differences through querying the probability of chickens transitioning from different states of colonization using a series of Bayesian models presented below.

## Model Development

In this section we discuss the general methodology behind all of our models. A general step-by-step process to model formulation is also presented in Box 1. Each model begins by classifying each of the datapoints into certain state labels. For example, at the simplest level each reading can be classified as either “State 1: Uncolonized” or “State 2: Colonized.” Other models may use more states to further distinguish colonizations by species or ST. After doing this, we are able to convey this classification data in the form of a matrix *S*[*c, t*] where *c* ∈ {1, 2, …, 200} is the index denoting which chicken is considered, and *t* ∈ {1, 2, …, 51} is the index denoting which week is considered. Therefore, each element of *S* will be a number conveying the state classification of that particular data point. For example, *S*[3, 7] = 1, would indicate that on week 7, chicken number 3 was classified as state 1; uncolonized. Because only 75 of the 200 chickens were tested at random each week, many of these matrix elements are undefined, and as such are marked as “NA.”

Box 1 **Model construction process.**

*1. Decide state classifications*.

Choose how data should be classified, and construct matrix *S* containing all state classifications for each data point.

*2. Decide formulation of transition matrix*.

Choose how model will define transition probabilities and dependencies.

*3. Run Bayesian model*.

Define prior probability distributions for model parameters. Program and run Bayesian model using JAGS, to acquire a posterior probability distribution for all model parameters defined in step 2.

*4. Assess convergence*.

Investigate model output to assure posterior distribution is well-constructed and has converged.

*5. Present results*.

Plot the transition probabilities, π_*i, j*_, and interpret the results.

Once the matrix is defined, each model uses a Bayesian process to find the transition probabilities between these states. Formally we seek the matrix π, where π_*i, j*_ = *P*(*S*[*m, n*] = *j*|*S*[*m, n* − 1] = *i*), for every *m* ∈ {1, 2, …, 200} and *n* ∈ {2, 3, …, 51}. In short, π_*i, j*_ is the probability that a chicken moves from state *i* to state *j* across a week. The exact choice of how to formulate the expressions is where our models vary, as different formulations are able to investigate different relationships governing these transition probabilities. For example, at the simplest level, we could define

(1) $$\begin{array}{l} \pi_{1,1}=\alpha_{1}\\ \pi_{1,2}=1-\pi_{1,1}\\ \pi_{2,1}=\alpha_{2}\\ \pi_{2,2}=1-\pi_{2,1} \end{array}$$

where we seek to find the values α_1_ ∈ [0, 1] and α_2_ ∈ [0, 1] that best fit the data *S*. Note that we have bounded π_*i, j*_ between 0 and 1, as each value represents a probability. Likewise each row of π must sum to 1, as these probabilities cover all transition possibilities. In the example of Equation (1) above, when starting from state 1, one can transition to state 2 (π_1,2_), or remain in state 1 (π_1,1_), hence π_1,1_ + π_1,2_ = 1. Different models below will use more complex definitions for π to explore the impact of time, density dependence, and chicken health on transitions between different states.

A Bayesian statistical model provides a way to iteratively deduce parameters of interest in regards to given data. The process is derived from Bayes' theorem:

(2) $$P(\theta |D)=\frac{P(D|\theta )P(\theta )}{P(D)},$$

where θ is the parameter/s we wish to discover, and *D* is the data provided. In short, Equation (2) reads that when starting from an initial, **prior**, belief in what values θ may take (*P*(θ)), one may obtain an updated, **posterior**, probability distribution for these possible values given some provided data (*P*(θ|*D*)). A more thorough introduction to Bayesian modeling is provided in Appendix 1. In our case, the parameters we seek, θ, are the ones used in our definition of π, such as α_1_ and α_2_ in the example above. The data, *D*, we use is the matrix *S*.

Below we present a series of case studies presenting our different models and their results. All models were run using JAGS (Plummer, 2007) from within R using the run.jags package (Plummer et al., 2016). All code used for the following models is made available at https://osf.io/m5yua/.

## Case Studies

### Model 1: Time Dependence

Our first model investigates how time affects the transition probabilities between states. Following the process outlined in Box 1, we choose to initially classify our data as one of two states: “state 1: uncolonized” and “state 2: colonized.”

To assess how the transition probabilities vary through time we must ensure that we define our transition probabilities such that they depend on time. One way would be to adapt Equation (1) above such that π_1,1_ was a function of α + β*t*. However, this would impose structure upon the transition probabilities, enforcing them to change linearly with time. Ideally a model formulation should allow as much freedom as possible to fit to the data. As such, we shall instead construct π as a three-dimensional array. In essence this means that each time period can be described by its own transition matrix. Formally we write this as,

(3) $$\begin{array}{l} \pi_{1,2,t}=\text{ilogit}(\alpha_{1}+C_{1}[t]),\\ \pi_{1,1,t}=1-\pi_{1,2,t},\\ \pi_{2,1,t}=\text{ilogit}(\alpha_{2}+C_{2}[t]),\\ \pi_{2,2,t}=1-\pi_{2,1,t}, \end{array}$$

for *t* ∈ {1, 2, …, 51}. Here ilogit() is the inverse logit function defined by ilogit(*x*) = $\frac{e^{x}}{1+e^{x}}$. This function is bounded between 0 and 1, scaling the argument so that our probabilities, π_*i,j,t*_ remain correctly bounded. The underlying theory is that we assume there is some mean probability for π_*i,j,t*_ across all *t*. These mean probabilities are described by α_1_ and α_2_. We then assume that, for each *t*, there is some “correction term” away from the mean unique to each week. These correction terms are captured by *C*_1_[*t*] and *C*_2_[*t*] for each *t*.

Now that we have decided on our model formulation, we move to step 3 and run the model to find the posterior distributions for α_1_, α_2_, *C*_1_, and *C*_2_. First we define our prior probability distributions for each of the model parameters. This distribution represents our initial assumptions on what value our variables may take, and is often informed by expert opinion. Since we do not have any initial assumptions on what values our variables may take, we use wide non-informative priors. For α_1_ and α_2_ we choose a prior distribution of *U*(0, 25) for each, a uniform distribution between 0 and 25. For *C*_1_ and *C*_2_, we wish each element of these vectors to be a small perturbation away from the mean of α_1_ or α_2_. As such, we would ideally have these elements drawn from a normal distribution with mean 0, and some, yet to be determined, standard deviation. This represents a hierarchical model formulation (discussed further in Appendix 1), where we instead define priors on the two standard deviations for these two normal distributions associated with *C*_1_ and *C*_2_. Following the advice of Gelman (2006) for non-informative improper priors, we use a uniform distribution between 0 and 50 for the prior distribution of each of these standard deviation parameters. The model was then run using two chains, with a burn-in period of 5,000 iterations, and then a final sample of 25,000 iterations to build the posterior distributions.

Convergence was considered well-achieved via investigation of the trace plots of the chains, the effective sample size (ESS) and Monte Carlo Standard Error (MCSE) of the variables. The Gelman-Rubin statistic, or “shrink factor,” is the most commonly used metric for convergence, with a value close to 1 signifying effective convergence. Heuristically, any shrink factor below 1.1 is considered by Kruschke (2014) to signify sufficient convergence. The presented model run resulted in a multivariate potential scale reduction factor (mpsrf) of 1.0059.

**Figure 4:**
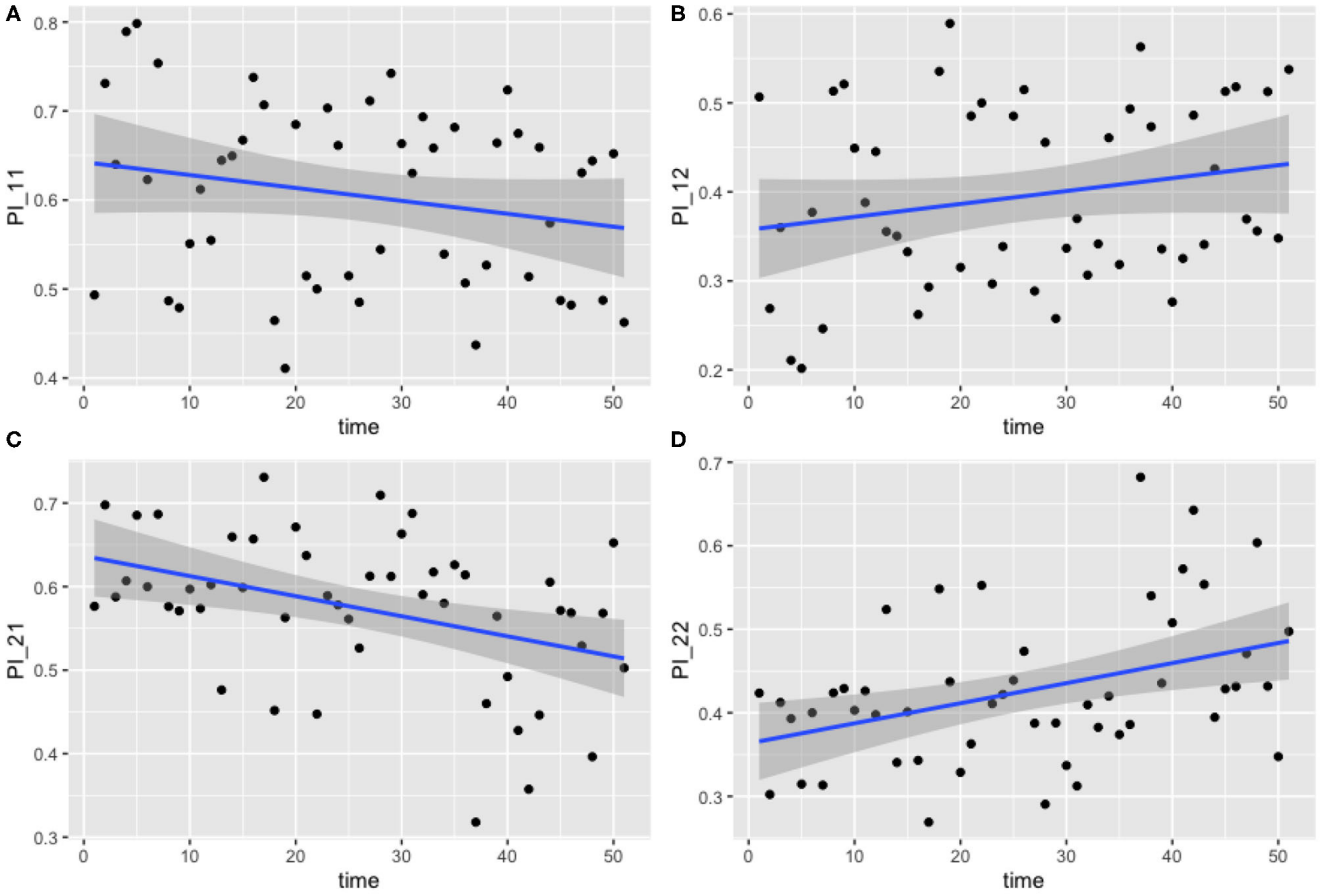
Transition probabilities between two states, “uncolonized” and “colonized.” Plots show **(A)** π_1,1,*t*_, **(B)** π_1,2,*t*_, **(C)** π_2,1,*t*_, and **(D)** π_2,2,*t*_ against time. Each point is the calculated transition probability for that time point. Also plotted is a linear regression against these points in blue, with a shaded region depicting the 95% confidence interval of the regression. **(C,D)** Are significant (*p* < 0.01).

The results for this model are presented below in Figure 4. The median values of the transition probabilities for (4A) π_1,1,*t*_, (4B) π_1,2,*t*_, (4C) π_2,1,*t*_, (4D) π_2,2,*t*_ are plotted, and a linear regression is fit to these outputs using the lm function in R. Fitting a general additive model (GAM) to these median values revealed that there was no significant model fit for higher order models, hence only linear regression fits are displayed. The probability of transitioning from state 1 (plots 4A and 4B) was not significantly correlated against time (*t*-test, *p* = 0.135), however transitions from state 2 (plots 4C and 4D) against time were statistically significant (*t*-test, *p* < 0.01).

These findings suggest that, as time progresses, colonized chickens become more likely to remain colonized, and similarly become less likely to clear such a colonization. Figures 4C,D show that, at the start of the experiment, colonized chickens would be more likely to clear the colonization the following week, but by the end of the experiment this had reduced to a probability of roughly 50%.

### Model 2: Species Dependence

For the next model we investigate transition differences between the two species present in the study: *C. jejuni* and *C. coli*. As such, this time we classify our data as belonging to one of three states; “state 1: uncolonized,” “state 2: colonized by *C. jejuni*,” and “state 3: colonized by *C. coli*.” Therefore our transition matrix will be of size 3 × 3. We define each row of the transition matrix by a 3-variable Dirichlet distribution (the multivariate generalization of the Beta distribution), ensuring each row sums to 1. As such, we infer the transition probabilities directly, using prior distributions of

$$\begin{array}{l} (\pi_{1,1},\pi_{1,2},\pi_{1,3})=\text{Dirichlet}(1,1,1),\\ (\pi_{2,1},\pi_{2,2},\pi_{2,3})=\text{Dirichlet}(1,1,1),\\ (\pi_{3,1},\pi_{3,2},\pi_{3,3})=\text{Dirichlet}(1,1,1). \end{array}$$

**Figure 5:**
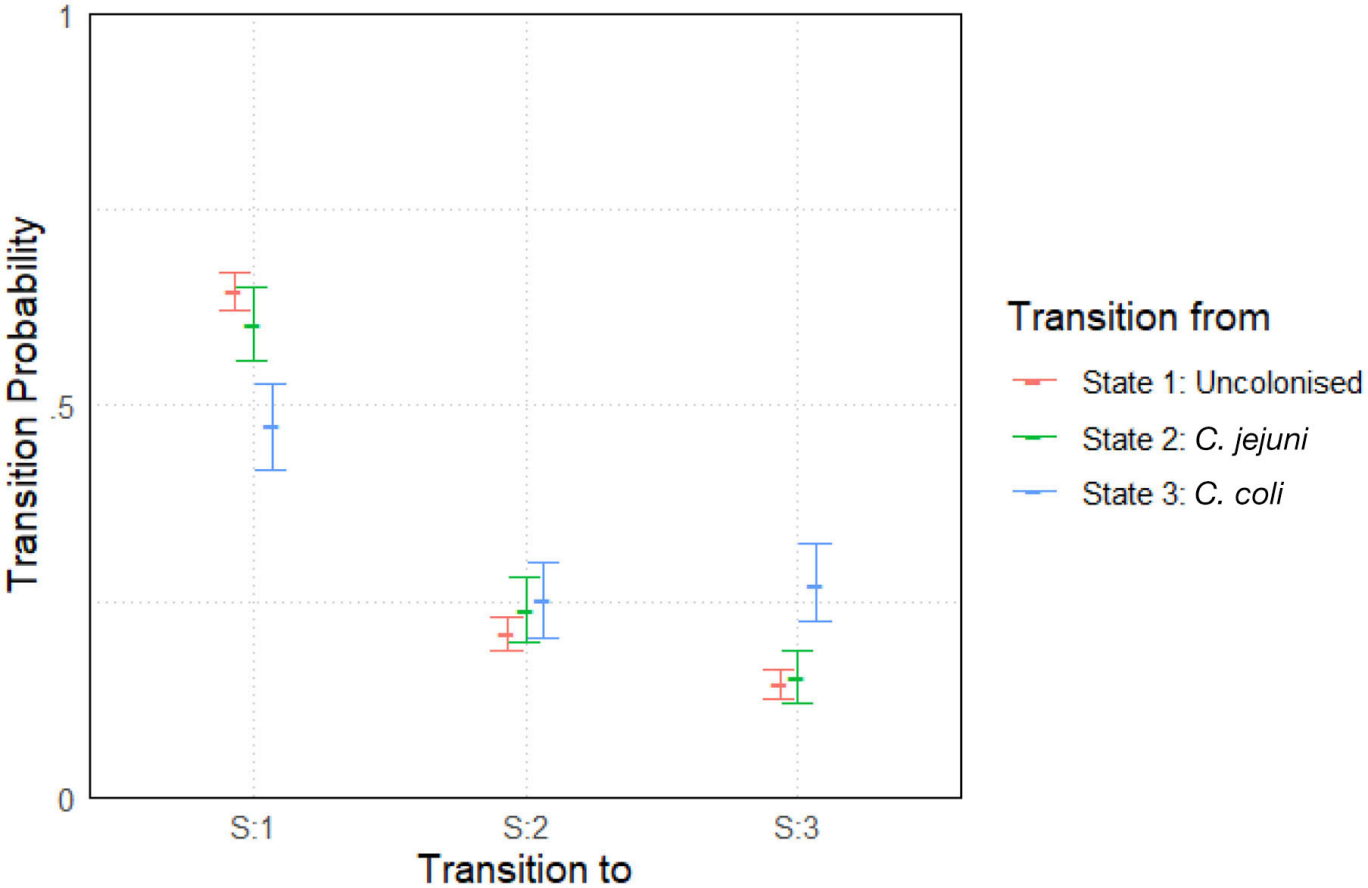
Transition probabilities between three states, “uncolonized,” “colonized by *C. jejuni*,” and “colonized by *C. coli*.” Plots show the median values of the posterior distributions and the 95% highest density intervals (HDIs).

The model was run with two chains and an initial burn-in period of 5,000 iterations. Posterior distributions were built from a sample of 10,000 iterations. Convergence was once again well-achieved with a mpsrf of 1.0035. The results are plotted below in Figure 5. Results show slight variations between species across the entire experiment. General transition probabilities from each state are very similar, however one can note that a chicken is more likely to be colonized by *C. coli* when transitioning from a state of already being colonized by *C. coli*. We also see that a chicken colonized by *C. coli* is less likely to transition to being uncolonized than a chicken colonized by *C. jejuni*.

### Model 3: Time and Species Dependence

We now combine the previous two models together, to investigate how the transitions between species alter across time. We once again therefore classify our data into three categories, as per the previous model.

We will be constructing a three-dimensional array once again for our transition probabilities, with each time period being described by a separate 3 × 3 transition matrix. To ensure each row of these matrices sums to 1, we start by framing the transition probabilities as an unbounded array *p*, before scaling these into our final array π. *p* is defined as

(4) $$\begin{array}{ll} p_{1,1,t}=\text{exp}(\alpha_{1}), & p_{1,2,t}=\text{exp}(\alpha_{2}+C_{1}[t]),\\ p_{1,3,t}=\text{exp}(\alpha_{3}+C_{2}[t]), & p_{2,1,t}=\text{exp}(\alpha_{4}),\\ p_{2,2,t}=\text{exp}(\alpha_{5}+C_{3}[t]), & p_{2,3,t}=\text{exp}(\alpha_{6}+C_{4}[t]),\\ p_{3,1,t}=\text{exp}(\alpha_{7}), & p_{3,2,t}=\text{exp}(\alpha_{8}+C_{5}[t]),\\ p_{3,3,t}=\text{exp}(\alpha_{9}+C_{6}[t]). \end{array}$$

The exponential function here assures that, like in our initial model, our α parameters will describe the average transition value across time, with the *C* parameters describing a small perturbation away from this mean. *C* values only need to be implemented on two probabilities in each row, as we will next scale these so that each row sums to 1, meaning that two free correction terms are sufficient to describe the distribution of the row. Our scaling is then performed like so,

(5) $$\begin{array}{l} \pi_{1,1,t}=\frac{p_{1,1,t}}{p_{1,1,t}+p_{1,2,t}+p_{1,3,t}},\;\pi_{1,2,t}=\frac{p_{1,2,t}}{p_{1,1,t}+p_{1,2,t}+p_{1,3,t}},\;\\ \pi_{1,3,t}=\frac{p_{1,3,t}}{p_{1,1,t}+p_{1,2,t}+p_{1,3,t}},\;\pi_{2,1,t}=\frac{p_{2,1,t}}{p_{2,1,t}+p_{2,2,t}+p_{2,3,t}},\\ \pi_{2,2,t}=\frac{p_{2,2,t}}{p_{2,1,t}+p_{2,2,t}+p_{2,3,t}},\;\pi_{2,3,t}=\frac{p_{2,3,t}}{p_{2,1,t}+p_{2,2,t}+p_{2,3,t}},\\ \pi_{3,1,t}=\frac{p_{3,1,t}}{p_{3,1,t}+p_{3,2,t}+p_{3,3,t}},\;\pi_{3,2,t}=\frac{p_{3,2,t}}{p_{3,1,t}+p_{3,2,t}+p_{3,3,t}},\\ \pi_{3,3,t}=\frac{p_{3,3,t}}{p_{3,1,t}+p_{3,2,t}+p_{3,3,t}}.\; \end{array}$$

We choose priors of *N*(0, 1000) for all our α values (normal distributions with mean 0 and standard deviation 1,000). Like the first model, we shall construct a hierarchical dependency such that our *C*_*i*_[*t*] are all drawn from a normal distribution for each *t*. Motivated by the correlation observed in the first model, we actually set these six *C*_*i*_ terms to all be drawn from a six-variable multivariate normal distribution, with mean (0, 0, 0, 0, 0, 0) and a covariance matrix as our parameter to be defined. JAGS requires the input of a precision matrix (the inverse of the covariance matrix) for its formulation of the multivariate normal distribution, so we set a prior distribution on the precision matrix of Wishart(*I*_6_, 6), where *I*_6_ is the 6 × 6 identity matrix.

**Figure 6:**
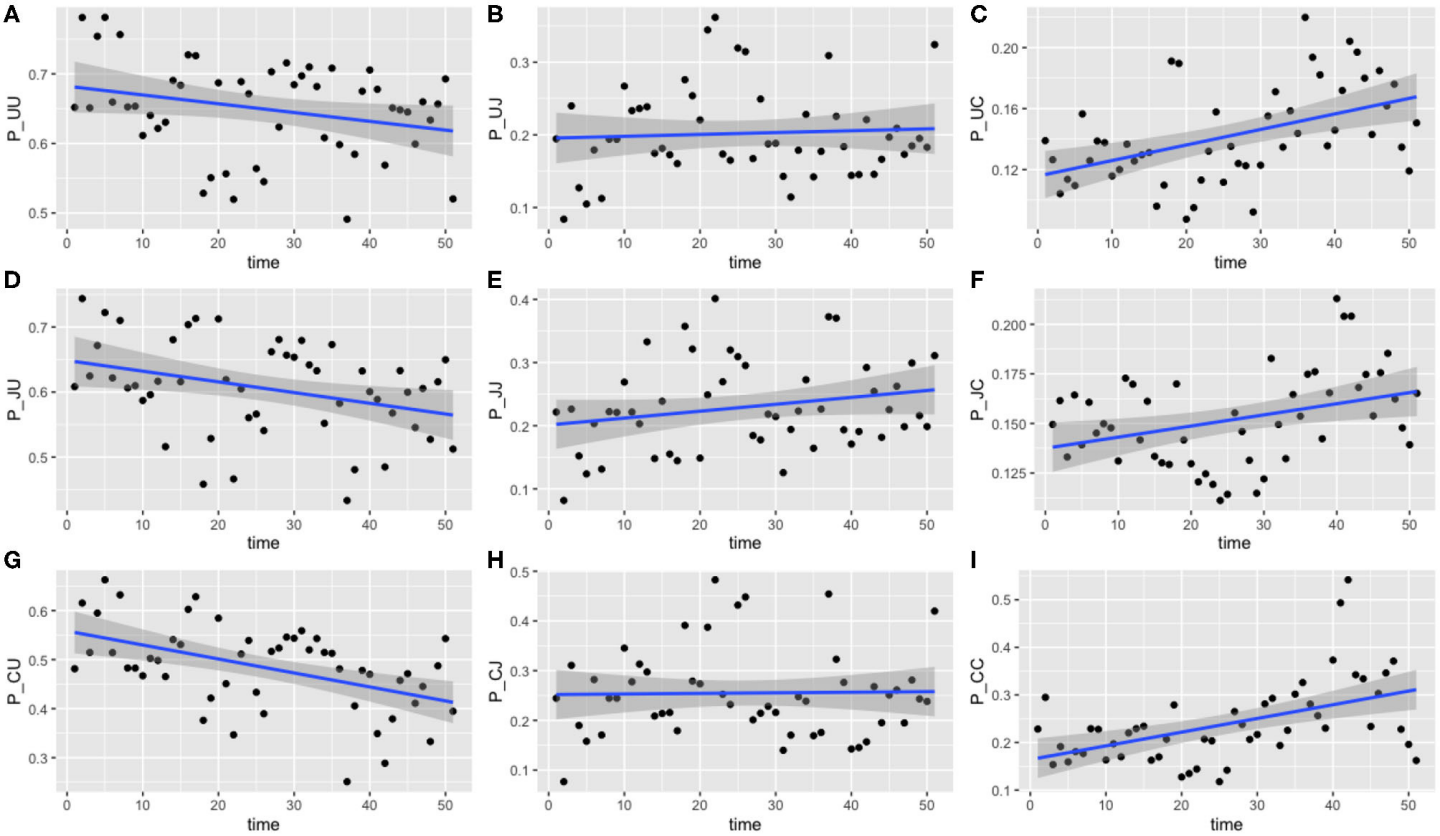
Transition probabilities between three states, “uncolonized,” “colonized by *C. jejuni*,” and “colonized by *C. coli*.” Plots show **(A)** π_1,1,*t*_, **(B)** π_1,2,*t*_, **(C)** π_1,3,*t*_, **(D)** π_2,1,*t*_, **(E)** π_2,2,*t*_, **(F)** π_2,3,*t*_, **(G)** π_3,1,*t*_, **(H)** π_3,2,*t*_, and **(I)** π_3,3,*t*_ against time. Each point is the calculated transition probability for that time point. Also plotted is a linear regression against these points in blue, with a shaded region depicting the 95% confidence interval of the regression. Five transition probabilities were found to be statistically significant for correlation against time: π_1,3,*t*_, π_2,1,*t*_, π_2,3,*t*_, π_3,1,*t*_, and π_3,3,*t*_ (*t*-tests, *p* < 0.0005, *p* < 0.05, *p* < 0.05, *p* < 0.0005, and *p* < 0.0005, respectively).

The model was run with two chains for an initial burn-in period of 5,000 iterations, and then a posterior distribution was built from a sample of 250,000 iterations, thinned at a rate of 1 in 5, meaning only 1 in every 5 iterations was used for the posterior distribution so as to reduce autocorrelation. Results are plotted below in Figure 6.

**Figure 7:**
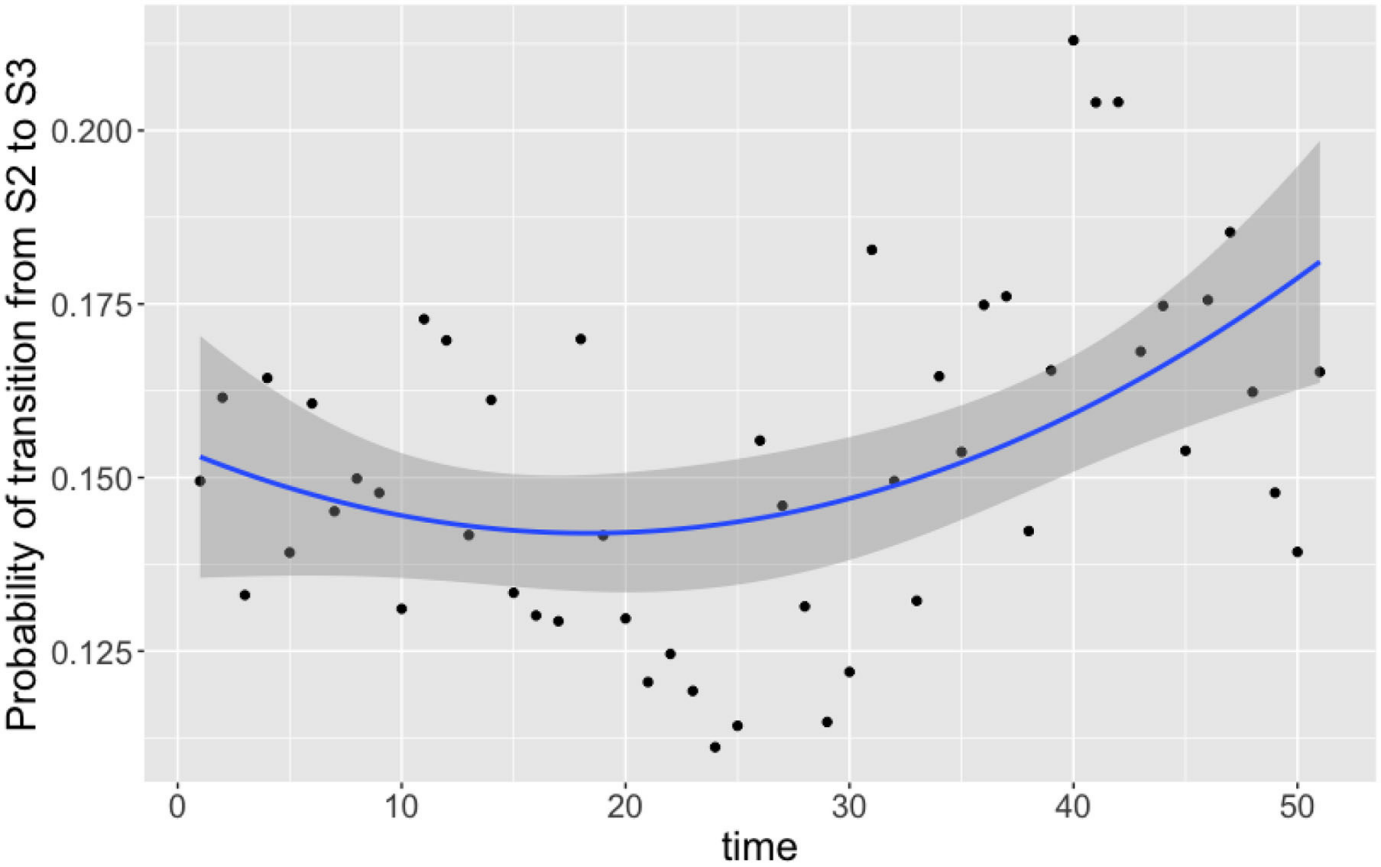
Transition probabilities between state 2 “colonized by *C. jejuni*” and state 3 “colonized by *C. coli*” against time. Each point is the calculated transition probability for that time point. Also plotted is a quadratic regression against these points in blue, with a shaded region depicting the 95% confidence interval. The transition probability was found to be statistically significant for correlation against time (*t*-test, *p* < 0.05).

Of the nine transition probabilities presented, five were found to be statistically significant for correlation against time when a linear regression was applied: π_1,3,*t*_, π_2,1,*t*_, π_2,3,*t*_, π_3,1,*t*_, and π_3,3,*t*_ (*t*-tests, *p* < 0.0005, *p* < 0.05, *p* < 0.05, *p* < 0.0005, and *p* < 0.0005, respectively). We once again see that transitions to a state of uncolonization reduce over time, however, whereas model 1 reported overall transitions to a state of colonization increasing, model 3 shows that only transitions to colonization by *C. coli* increase over time. Given the spread of the data in Figure 6, we also tested for statistical significance against a quadratic regression. A quadratic fit would be a strong argument for the existence of seasonal variation, by capturing a difference in the middle of the time series as the time axis moves to summer, before returning to winter. Recall again that this time period plotted is in weeks from February 2004 to February 2005. Only one transition probability was found to be statistically significant however, the transition from colonization by *C. jejuni* to *C. coli*, π_2,3,*t*_ (*t*-test, *p* < 0.05). This quadratic regression is presented in Figure 7 below. This would correlate with the behavior observed in Figures 2, 3, whereby *C. jejuni* appears to be most prevalent in the summer, and *C. coli* most prevalent in the winter (similarly to model 1, fitting a general additive model (GAM) to these median values revealed that there was no significant model fit for higher order models, hence only linear regression fits, and the one quadratic regression fit, are displayed).

### Model 4: ST Perseverance

For this model, we extend model 2 to now capture species-specific ST perseverance within a chicken. To do this, we re-classify the data into five different states: “S1: uncolonized,” “S2: new *C. jejuni* ST,” “S3: same *C. jejuni* ST as previous week,” “S4: new *C. coli* ST,” and “S5: same *C. coli* ST as previous week.” To further clarify the meaning of state 2 and state 4, we mean a ST of either *C. jejuni* or *C. coli* that was not present in the previous week for the chicken in question. For example, if one chicken had the following colonization data for 10 days: {“Uncolonized,” “Colonized by *C. coli* ST 1089,” “Colonized by *C. coli* ST 1090,” “Colonized by *C. coli* ST 1090,” “NA,” “Colonized by *C. coli* ST 1090,” “Colonized by *C. jejuni* ST 958,” “Colonized by *C. jejuni* ST 958,” “Colonized by *C. jejuni* ST 1257,” “Uncolonized},” then this row of ten would be classified as { 1, 4, 4, 5, NA, 4, 2, 3, 2, 1 }. Because, by definition, one can only transition to state 3 from state 2 or state 3, we can fix π_1,3_ = π_4,3_ = π_5,3_ = 0, and likewise for transitions to state 5: π_1,5_ = π_2,5_ = π_3,5_ = 0. The non-zero transition probabilities can then be calculated by drawing each row from a 3 or 4 variable Dirichlet distribution. Formally we set a prior on each row of,

(6) $$\begin{array}{l} \;(\pi_{1,1},\pi_{1,2},\pi_{1,4})\sim \text{Dirichlet}(1,1,1),\\ \left(\pi_{2,1},\pi_{2,2},\pi_{2,3},\pi_{2,4}\right)\sim \text{Dirichlet}(1,1,1,1),\\ \left(\pi_{3,1},\pi_{3,2},\pi_{3,3},\pi_{3,4}\right)\sim \text{Dirichlet}(1,1,1,1),\\ \left(\pi_{4,1},\pi_{4,2},\pi_{4,4},\pi_{4,5}\right)\sim \text{Dirichlet}(1,1,1,1),\\ \left(\pi_{5,1},\pi_{5,2},\pi_{5,4},\pi_{5,5}\right)\sim \text{Dirichlet}(1,1,1,1). \end{array}$$

**Figure 8:**
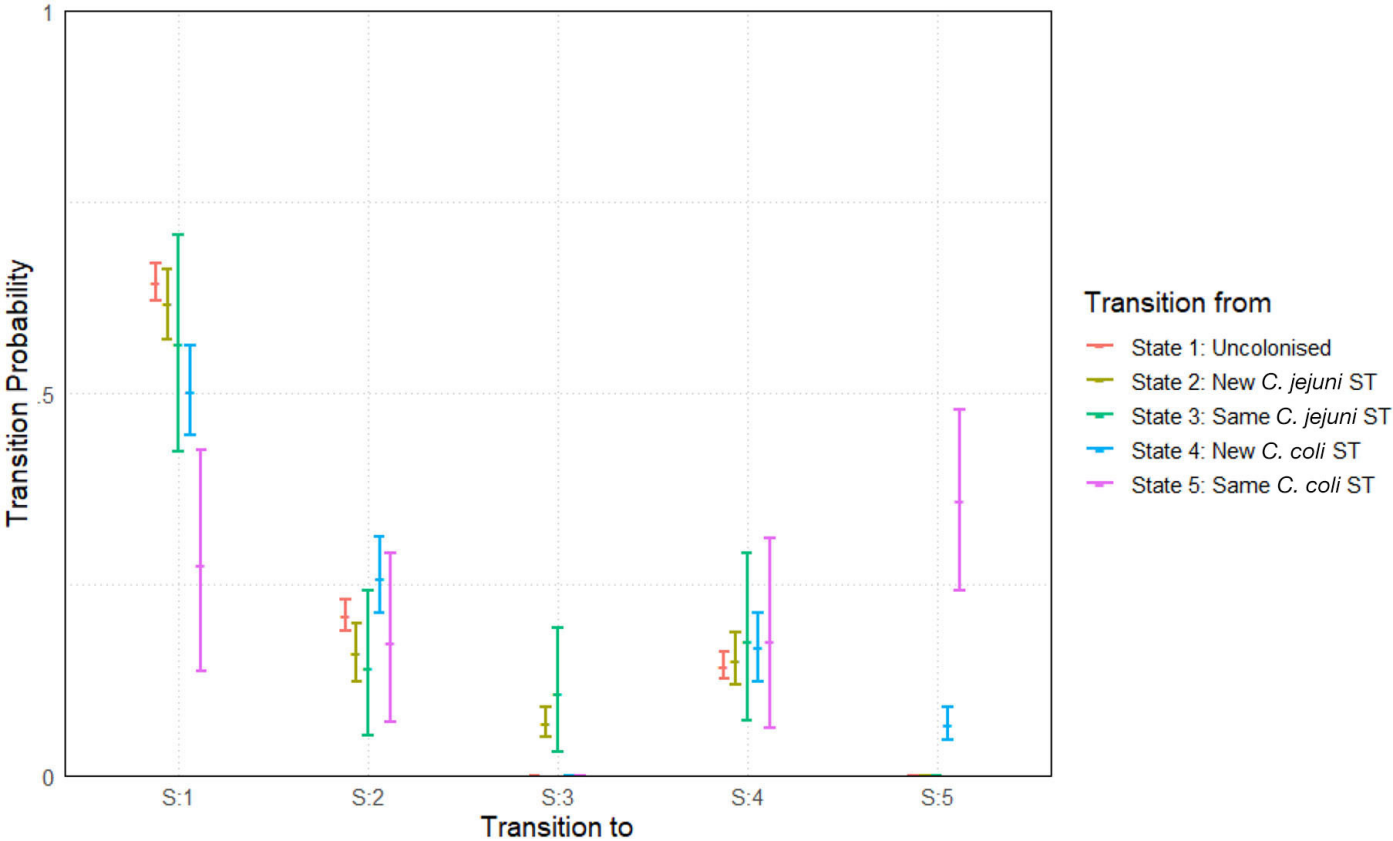
Transition probabilities between five states, “uncolonized,” “colonized by a new *C. jejuni* ST,” “colonized by the same *C. jejuni* ST as previously,” “colonized by a new *C. coli* ST,” and “colonized by the same *C. coli* ST as previously.” Plots show the median values of the posterior distributions and the 95% highest density intervals (HDIs).

The model was run with two chains for a burn-in period of 5,000 iterations before building posteriors from a final sample of 10,000 iterations. Chains were well-mixed and convergence well-achieved with an mpsrf of 1.0037. Results are plotted below in Figure 8.

The most notable difference is seen in the perseverance of *C. coli* STs compared to *C. jejuni* STs. Comparing columns 3 and 5 of Figure 8, we see that, a colonization by a new ST of either *C. coli* or *C. jejuni* has a roughly equal chance of persevering to the next week. However, once a ST has carried over for 1 week, *C. coli* colonizations are then considerably more likely to further persist for later weeks. In fact, a repeated instance of colonization by a *C. coli* ST (state 5) is more likely to continue in subsequent weeks than to transition to any other state (seen by comparing the pink lines in Figure 8). Comparing also columns 2 and 3 of Figure 8, we see that transitions to colonizations by new hboxtextitC. coli/*C. jejuni* STs are roughly comparable, meaning that the primary difference we observe between the two species is in perseverance as opposed to infectivity.

### Model 5: Chicken Dependence

Whereas model 1 considered how transition probabilities vary across time, we now consider how transition probabilities vary across different chickens. We follow a very similar framework to model 1, beginning by classifying all data as one of two states: “S1: uncolonized” or “S2: colonized.” We then, like model 1, consider some average transition probability that each chicken is close to, and then consider some small “correction term” unique to each chicken, which may make them more or less likely to transition to a certain state. Formally, we write,

(7) $$\begin{array}{l} \pi_{1,2,t}=\text{ilogit}(\alpha_{1}+C_{1}[c]),\\ \pi_{1,1,t}=1-\pi_{1,2,t},\\ \pi_{2,1,t}=\text{ilogit}(\alpha_{2}+C_{2}[c]),\\ \pi_{2,2,t}=1-\pi_{2,1,t}, \end{array}$$

for *c* ∈ {1, 2, …, 200}. We set a non-informative prior distribution for α_1_ and α_2_ of *N*(0, 1000). Our chicken correction terms, *C*_1_[*c*] and *C*_2_[*c*], are each drawn from a two-variable multivariate normal distribution for each *c*, with mean (0, 0) and covariance matrix to be calculated. Like described in model 3, we therefore set a prior distribution on the precision matrix for this multi-variate normal distribution of Wishart(*I*_2_, 2), where *I*_2_ is the 2 × 2 identity matrix.

The model was run with two chains for an initial burn-in period of 20,000 iterations, before posteriors were then constructed from a sample of 50,000 iterations. Convergence was well-achieved, with all chains well-mixed and all parameters sampled with a high ESS and MCSE < 0.01. The mpsrf was unable to be calculated due to the high number of stochastic nodes, however there were no signs to suggest invalid convergence.

**Figure 9:**
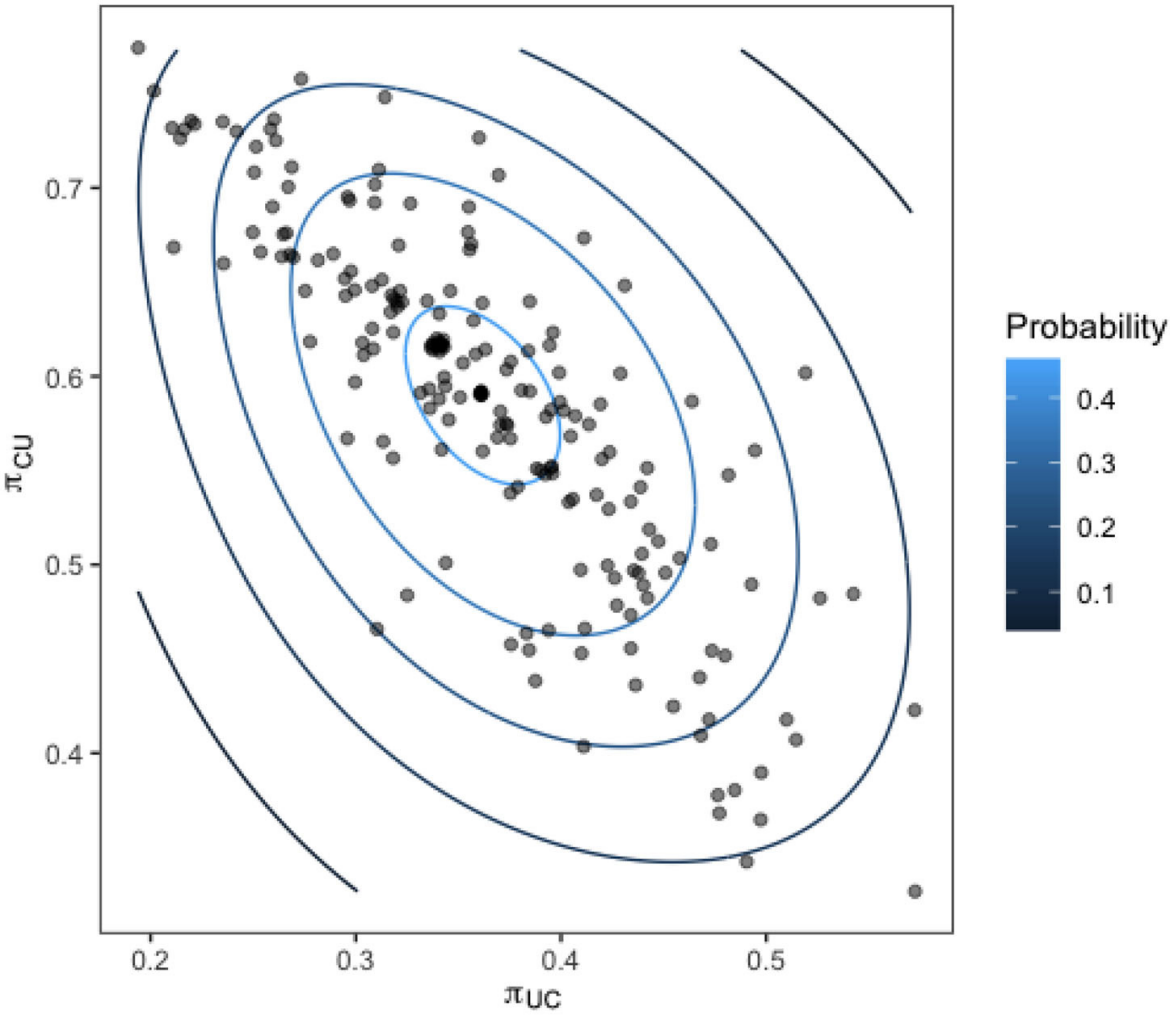
Transition probabilities for each bird in the flock from a state of being colonized to uncolonized (y-axis) against the transition probability from uncolonized to colonized (x-axis). Contours show the fit of a multivariate normal distribution to the output.

Upon calculating our transition probabilities for each bird, we plot the values for π_1,2_ against the value of π_2,1_ for each bird and investigate the correlation. Figure 9 shows these results overlaid with a contour of the associated multivariate normal distribution, indicating the probability density of the transition probabilities for the flock.

The strong linear relation observed reveals the presence of distinct sub-groups within the flock of birds who are colonized often, and those who are colonized very rarely.

### Model 6: Chicken and Species Dependence

We now alter the previous model to consider the differences in transition between species of *Campylobacter* across all birds. As such, the data is instead classified into the three states: “state 1: uncolonized,” “state 2: colonized by *C. jejuni*,” and “state 3: colonized by *C. coli*.” This model is formulated the same way as in model 3 above. The transition probabilities follow the same structure as Equations (4) and (5), except that our correction terms *C*_*i*_[*c*] are corrections for each chicken in the flock (*c* ∈ {1, 2, …, 200}) as opposed to each time step. As such we craft a 3 × 3 transition matrix for each chicken. A prior distribution of *N*(0, 1000) is used for each α_*i*_ parameter, and the six chicken correction terms, *C*_*i*_[*c*] are drawn from a six-variate multivariate normal distribution for each *c*, with mean (0, 0, 0, 0, 0, 0) and a precision matrix as a parameter to find. The prior distribution for this precision matrix is Wishart(*I*_6_, 6), where *I*_6_ is the 6 × 6 identity matrix.

The model was run with two chains for an initial burn-in period of 10,000 iterations, before posterior distributions were constructed from a sample of 50,000 iterations, thinned at a rate of 1 in 25, meaning only one iteration was kept in every 25.

**Figure 10:**
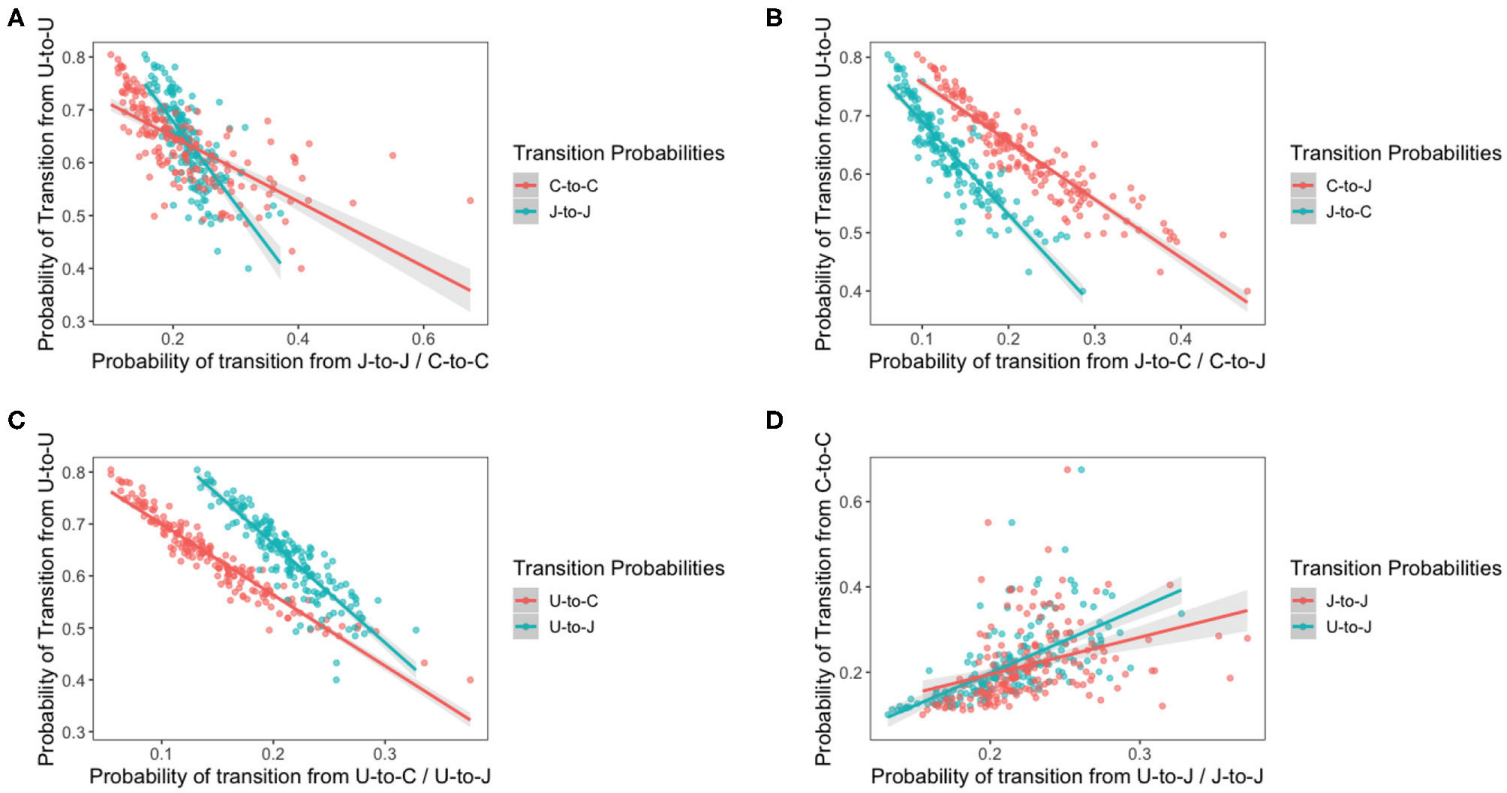
Transition probabilities for a three state system. In these plots, “U” refers to being uncolonized, “J” refers to colonization by *C. jejuni* and “C” refers to colonization by *C. coli*. Each of the points is the transition probability for a specific bird within the flock. Linear regression fits are plotted with a shaded region representing the 95% confidence intervals of the regression. All regressions were statistically significant (*t*-test *p* < 0.0001). **(A)** The transition probabilities of J-to-J and C-to-C against the transition probability of U-to-U. **(B)** The transition probabilities of C-to-J and J-to-C against the transition probability of U-to-U. **(C)** The transition probabilities of U-to-J and U-to-C against the transition probability of U-to-U. **(D)** The transition probabilities of J-to-J and U-to-J against the transition probability of C-to-C.

The idea of this model is to assess how bird variation affects the transition of each species of *Campylobacter*. The previous model revealed the existence of variation in bird resistance to colonization throughout the flock. Figure 10 below plots the result of multiple transition probabilities against one-another. Each point on the graphs represents the transition probabilities for a specific chicken. Plots 10A–C use π_1,1,*c*_, the transition from uncolonized to uncolonized as the y-axis. This acts as a rough metric for “bird resilience to colonization,” as the more resistant birds are more likely to continue being uncolonized. As such plots 10A–C depict how transitions related to each species vary according to host bird susceptibility. Plot 10D uses π_3,3,*c*_, the transition from *C. coli* to *C. coli* as the y-axis, to compare how the perseverance of *C. coli* affects the colonizing ability of *C. jejuni*. Linear regressions are fit to all plots in Figure 10, and all were found to be statistically significant (*t*-test, *p* < 0.0001).

Interestingly, the gradients of all the shifting transition probabilities are different between species, confirming that, indeed, the transition probabilities of each species varies differently across chickens. We see that the probability of a species persisting, unsurprisingly increases as bird susceptibility increases, but curiously our linear regressions for each species overlap. This result indicates that, in the more resilient birds, *C. coli* is less likely to persevere than *C. jejuni* colonizations, however the inverse is seen in the more susceptible birds.

It is interesting to note that the gradient of the lines in each plot are distinctly different from one another, highlighting how each species responds differently to variations in host bird health.

### Model 7: Chicken and Density Dependence

This model builds on model 5 by now considering how transition probabilities are affected by the number of total colonizations in the previous week. *Campylobacter* is known to be transmitted via the fecal-oral route between chickens, so it seems likely that a higher density of colonizations 1 week will cause an increased number of colonizations the following week. We classify our data into two states, uncolonized and colonized.

The model formulation is then as follows,

(8) $$\begin{array}{l} \pi_{1,2,c,t}=\text{ilogit}\;(\alpha_{1}+C_{1}[c]+\beta_{1}\left(\sum_{i=1}^{51}\frac{S[i,t]-1}{N_{t}}\right))\text{​},\\ \pi_{1,1,c,t}=1-\pi_{1,2,c,t},\\ \pi_{2,1,c,t}=\text{ilogit}\;(\alpha_{2}+C_{2}[c]+\beta_{2}\left(\sum_{i=1}^{51}\frac{S[i,t]-1}{N_{t}}\right))\text{​},\\ \pi_{2,2,c,t}=1-\pi_{2,1,c,t}, \end{array}$$

where *N*_*t*_ is the number of birds that data is available for at time *t*. Here, as with previous models, α_*i*_ represents some mean transition probability that all birds are clustered around, and *C*_*i*_[*c*] represents the slight correction for each bird *c*. Recall that the matrix *S* is populated by elements “1” denoting uncolonized and “2” denoting colonized. Therefore, the expression *S*[*i, t*]−1 for every *i* and *t* shifts this to instead be captured as “0” signifying uncolonized, and “1” signifying colonized. Therefore, the expression $\overset{51}{\underset{i=1}{\sum }}S\left[i,t\right]-1$ will be a tally of exactly how many birds are recorded as being colonized at time *t*. Therefore, the expression $\overset{51}{\underset{i=1}{\sum }}\frac{S\left[i,t\right]-1}{N_{t}}$ conveys the exact proportion of how many birds are currently colonized. Note the use of *N*_*t*_ as, for most weeks 75 birds are recorded for every *t*, however, as can be seen in Figure 1, occasionally a few more or less were recorded each week. Note however, that during the Bayesian modeling process, values for each element of *S* will be imputed in the process, meaning that we can choose to measure our density dependence using either just the provided data, or also the imputed data. There are merits to both approaches, and so results are included for both below. Here β_*i*_ are parameters signifying the strength of the density dependent effect.

**Figure 11:**
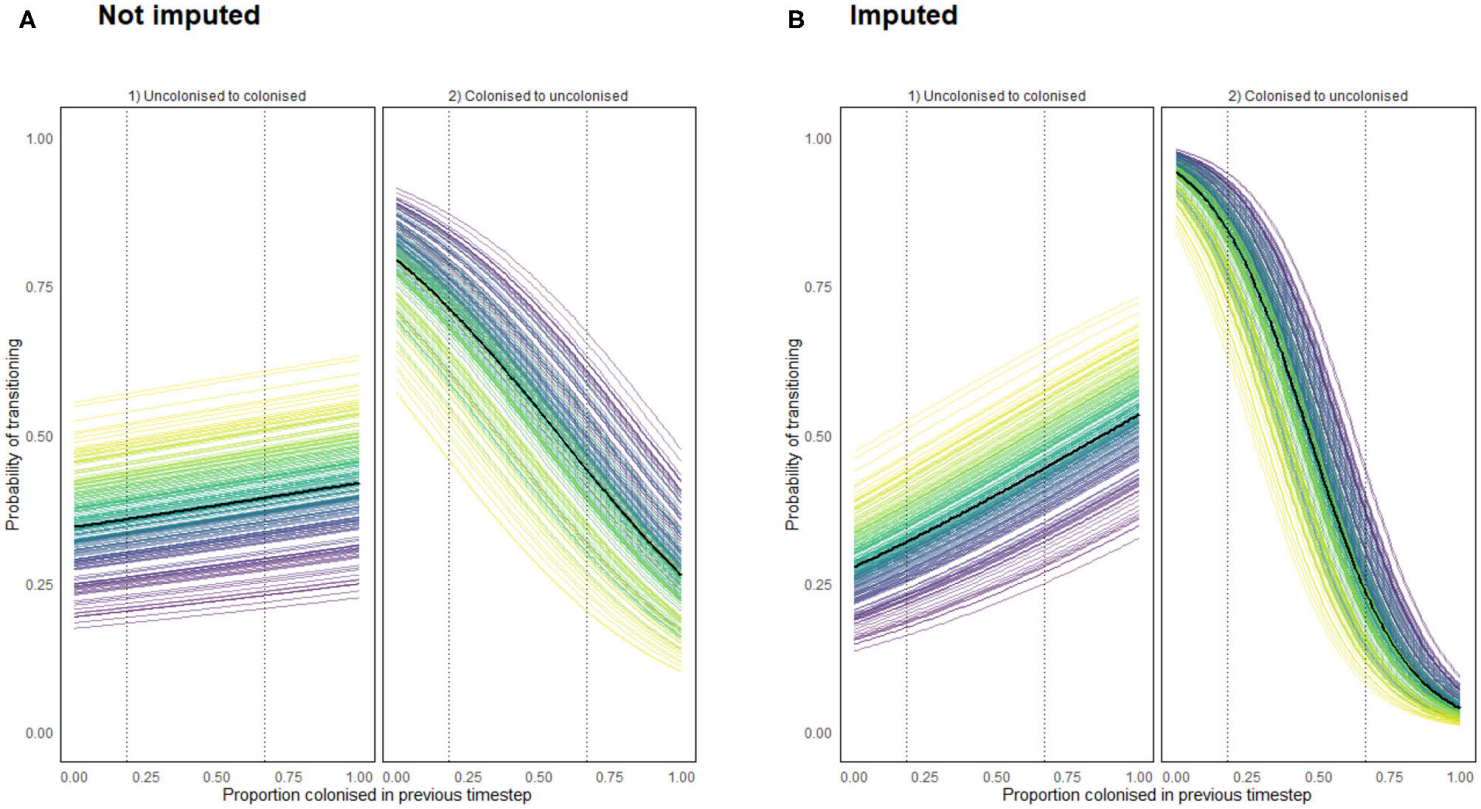
Transition probabilities from a state of uncolonization (by *Campylobacter*) to colonization, and from colonization to uncolonization using a density dependent model programmed using **(A)** recorded data **(B)** recorded and imputed data. Each colored line represents the transition probabilities for a single chicken, with a black line depicting the flock mean. Dotted lines show the region for which data was available for such a flock colonization proportion.

The model was initialized with prior distributions of *N*(0, 1000) for all α_*i*_ and β_*i*_ parameters. The chicken corrections terms *C*_*i*_[*c*] were, like above, drawn from a multivariate normal distribution of mean (0,0) whose precision matrix we seek. The precision matrix was initialized with a prior distribution of Wishart(*I*_2_, 2) where *I*_2_ is the 2 × 2 identity matrix. The model was run with two chains for an initial burn-in period of 6,000 iterations and then posterior distributions built from a sample of 25,000 iterations. This was done twice with two variations of the model. One where density dependence is calculated from provided data, and one with the addition of imputed data. The posterior distributions of our model parameters were used to simulate the transition probabilities for each flock across a full range of total flock prevalences, i.e., using the median values for α_*i*_, β_*i*_, *C*_*i*_ and the precision matrix, we are able to build the functions

(9) $$\pi_{1,2,c}=\alpha_{1}+C_{1}[c]+\beta_{1}D,$$

(10) $$\pi_{2,1,c}=\alpha_{2}+C_{2}[c]+\beta_{2}D,$$

for any value *D* ∈ [0, 1], for each chicken *c*. The results of these functions for both the imputed and non-imputed density models are presented below in Figure 11. The data only record flock colonizations proportions ranging from 0.1818 to 0.6667, so dotted lines are placed in Figure 11 to show the range beyond which the result was further imputed.

Importantly Figure 11 confirms that density dependence is apparent within the flock. This was an important result to capture to reinforce the findings of model 6. It confirms that birds are influenced by the colonization prevalence of the flock, suggesting that the more resilient birds truly are less likely to become colonized, as opposed to just never becoming exposed to particularly virulent STs. Of interest here is that the probability of clearing colonization (transitioning to uncolonized) is affected far more by flock prevalence proportion than the probability of becoming colonized.

## Discussion

An improved understanding of the transmission dynamics of *Campylobacter* among and within poultry flocks in the commercial environment is essential for the design of effective intervention strategies to reduce levels of human disease. Here, these dynamics were evaluated within a flock of broiler-breeder chickens through a series of seven models, each constructed to investigate and answer a specific question. The analyses demonstrated the extent to which data can capture and describe multiple underlying dynamical behaviors, when queried with modeling approaches.

A number of the analyses were consistent with the existence of a “*Campylobacter* super-shedder” state within the flock, with other “resilient” birds that rarely or never transmit *Campylobacter* (Models 1, 5, 6, and 7). These resilient birds persisted even with high levels of colonization amongst the remainder of the flock (Model 7). The existence of super-shedders, is well-documented for *Salmonella* colonization in chickens (Gopinath et al., 2012; Menanteau et al., 2018) and a small number of studies indicate varied shedding levels of *Campylobacter* amongst cattle (Rapp et al., 2012); however, although the models presented here were developed from field data, there is otherwise a paucity of published evidence for a *Campylobacter* super-shedder status in chickens. This observation warrants further investigation, as the concept of individual colonization-resistant birds within a flock raises the potential of alternative approaches to controlling *Campylobacter* colonization in chicken flocks, e.g., those based on feed and/or probiotics. Small-scale experiments have shown that probiotic treatment can reduce *Campylobacter* bacterial load in individual birds (Willis and Reid, 2008; Ghareeb et al., 2012), but the wider impact in flock transmission dynamics is poorly understood. Numerical modeling approaches have, however, highlighted the growth rate of competing bacteria as the most powerful factor in reducing the spread of *Campylobacter* (Rawson et al., 2019).

The models further indicated that individual bird status was more important than *Campylobacter* strain in determining the dynamics of flock colonization (Models 6 and 7) and, to our knowledge, this is the first time this has been shown. Most (26/39, 66.7%) of the *Campylobacter* sequence types (STs) observed have been isolated from other chickens (Colles et al., 2015), which is consistent with them being competent at colonizing chicken hosts, although there is still potential for competition, for example over space or metabolic advantage. Whilst *Campylobacter* is generally considered to be a commensal of the chicken gut, there is evidence that in some breeds there is a prolonged inflammatory response, damage to the gut mucosa and diarrhea (Humphrey et al., 2014). Our results are consistent with the colonization of a chicken flock by *Campylobacter* being multi-factorial process, and the health and welfare of individual birds should be considered alongside *Campylobacter* strain type. The immune response of chickens has been shown to be impacted by welfare measures, such as stocking density (Guardia et al., 2011; Gomes et al., 2014), food withdrawal, and heat stress (Burkholder et al., 2008). Consequently these results suggest an additional incentive to uphold good bird welfare, as only a small sub-population of susceptible birds can have a large impact on the colonization status of the whole flock. The birds were weighed on two occasions through the study, but no correlation was found between weight and *Campylobacter* shedding rate.

Having confirmed the existence of variation in bird transition probabilities, the possible impact of such variation on the proliferation of *Campylobacter* STs was investigated. Using a previously-published stochastic differential equation model of *Campylobacter* population dynamics within a broiler flock (Rawson et al., 2019), two variant scenarios were explored: (i) one simulating a homogenous flock of chickens; and (ii) another simulating variation in immune response. The simulations demonstrated that demographically equal strains of *Campylobacter* could be sustained at broadly different levels across the flock as a consequence of bird immune response (Appendix 2). This is a random process, in that whichever strain is initially acquired by a super-shedder is then shed in large amounts into the environment, increasing the likelihood of colonizing other birds in the flock. This result implies that the observation that some STs persist at higher levels than others in the flock, is likely due to the variation in bird transition probabilities, as opposed to phenotypic differences between STs. For example, ST 958 may appear more than ST 45 (Figure 2), not because it has a competitive advantage, but because it was initially ingested by super-shedders. Indeed, upon examination of the first recorded appearance of specific STs, the STs that would appear most frequently throughout the experiment were first observed in the most susceptible birds. Likewise the STs that appeared to die out were first observed in the more resilient birds; however, as only 75 out of 200 birds were sampled each week the exact date of when a ST first occurred cannot be determined.

With respect to the two species, there was evidence that *Campylobacter coli* was shed by individual birds more consistently over time compared to *Campylobacter jejuni*, with some indication that *C. coli* was more prevalent in the winter and *C. jejuni* more prevalent in summer (Models 2, 3, and 4). These results should be interpreted with caution since, although the number of birds from which *Campylobacter* was detected was variable from week to week, single colony picks (i.e., one *Campylobacter* isolate per bird) were used, meaning the two variables were not entirely independent in this instance.

Human incidence of campylobacteriosis has been shown to vary in a repeated pattern each year (Nylen et al., 2002), which numerous studies have correlated with a similar pattern observed in broiler house colonization rates (Kapperud et al., 1993; Patrick et al., 2004; Jore et al., 2010), an observation disputed by other studies (Humphery et al., 1993). Despite this, there was no effect of seasonality on *Campylobacter* spp. shedding rate detected by the modeling approaches here (Models 1 and 3, Figures 4, 6), or in the original study examining local environmental variables (Colles et al., 2015). This lack of seasonality could be due to the different housing conditions and diet provisions between broiler and breeder flocks (Leeson and Summers, 2010). Breeder flocks have also been shown to shed smaller amounts of *Campylobacter* than commercial broilers (Cox et al., 2002).

The age of the flock has been shown to be an important factor associated with increasing *Campylobacter* strain diversity. *C. coli* are more commonly isolated from older broiler flocks and may be over-represented in this broiler-breeder flock in comparison to commercially housed broilers, who live for typically 5–10 weeks before slaughter. The finding, of increased colonization duration as time progressed (Model 3), primarily by *C. coli*, was most likely due to the increased flock prevalence resulting in a positive feedback loop, whereby more *Campylobacter* is being shed into the environment by colonized birds, and then further ingested by the other birds in the flock before they are able to clear colonization. Biologically, there is some indication that *C. coli* differs from *C. jejuni* based upon its genomic structuring, with the majority of farm animal related *C. coli* isolates grouping into the large ST-828 clonal complex, and *C. jejuni* isolates forming around 40 complexes. The shorter colonization periods by *C. jejuni* (Models 4 and 6, Figures 8, 10) potentially reflects a stronger immune response from the host leading to control or clearance of STs, although this did not preclude colonization by other *C. jejuni* (or *C. coli*) STs. Due to the inherent nature of sampling, it is not possible to know if strains were cleared altogether, or if multiple *Campylobacter* strains colonized birds simultaneously, although the modeling methods used in this study were chosen to take this uncertainty into account as far as possible. These results further highlight the importance of individual bird responses in determining flock-wide prevalence, and further ecological competition hierarchy modeling could be applied to the same data to assess the impact this phenomenon has on ST population stability across time. If structured hierarchical competition could be verified, this would support our contention that flock-wide defensive strategies should be conducted at an individual bird level.

We also stress the importance of our final model in not just investigating the weekly bacterial prevalence turnover, but how it further substantiates the importance of bird-to-bird transmission. Without this one could argue from our earlier results that more resilient birds were simply the ones who did not ingest a more invasive ST. Figure 11 shows the influence of flock colonization proportion on transition probabilities. Most notably we see that the transition from uncolonized to colonized is affected less by total colonization prevalence than the transition from colonized to uncolonized. This means that in a highly colonized flock, uncolonized birds still have a possibility to not become colonized, while those who are already colonized will be far less likely to then clear their colonization. This would likely be caused by the immune system of currently uncolonized birds being just as likely as previously to prevent an initial colonization, but currently colonized birds will be more likely to add to their current bacterial load by ingesting more *Campylobacter* and reduce their likelihood of recovery.

In conclusion, these analyses have highlighted the diversity of individual bird response to bacterial challenge, and how this range of responses can be a key driver of *Campylobacter* prevalence dynamics. It is now important to find an observable metric that correlates with the resilience of a bird to colonization. If it were possible to identify “super-shedder” birds on the farm, targeted interventions could be instituted to improve their health, or to better inform industry of how to raise broiler flocks with low rates of shedding within a flock. Such super-shedders will amplify the prevalence of *Campylobacter* within a flock, leading to rapid colonization. Now that we have highlighted the critical role that bird health plays, future work must elucidate how one may act to help prevent the emergence of super-shedders within the flock.

## Data Availability Statement

The original contributions presented in the study are publicly available. This data can be found here: https://osf.io/m5yua/. Further inquiries can be directed to Thomas Rawson, thomas.rawson@zoo.ox.ac.uk.

## Author Contributions

FC collected the data. TR, RP, MM, and MB conceived the study. TR and RP built the models and wrote all associated code. TR wrote the manuscript. MD, FC, and MB supervised the project. All authors reviewed the manuscript.

## Conflict of Interest

The authors declare that the research was conducted in the absence of any commercial or financial relationships that could be construed as a potential conflict of interest.
