# Supplementary material for: A Mathematical Modeling Approach to Uncover Factors Influencing the Spread of Campylobacter in a Flock of Broiler-Breeder Chickens
Source: Front Microbiol. 2020 Oct 28;11:576646. doi: 10.3389/fmicb.2020.576646 (PMC7655537; doi:10.3389/fmicb.2020.576646)
Supplement: Supplementary file 1 [file Data_Sheet_1.pdf]

A Appendices

A.1 Appendix 1 - Bayesian Statistics

This brief section aims to convey the basic principles of Bayesian statistics, and familiarise the reader with the terminology that is be used throughout the manuscript. For an in-depth explanation, I recommend the text by Kruschke (2014)[37].

Bayesian statistics is derived wholly from the relationship defined by Bayes' theorem,

$$P(\theta|D) = \frac{P(D|\theta)P(\theta)}{P(D)}. \tag{11}$$

If we consider θ as some statistical parameter we wish to infer, and D as some data informing the parameter, then equation (1) expresses that the probability distribution for our value of θ, given our dataset ($P(\theta|D)$), is proportional to the **likelihood** of such data ($P(D|\theta)$) multiplied by the probability distribution of θ free of any data ($P(\theta)$).

Spoken plainly, one starts with a **prior** probabilistic understanding of the values θ, often informed by expert opinion, and by utilising relevant data, D, we update our belief in the values θ may take, producing a new **posterior** distribution. Mnemonically, if we wished to calculate the probability that a flipped coin will land heads up, we may have a **prior** belief that the coin is fair. However, upon observing a data set of 5 coin flips, all of which produced heads, we may update our **posterior** belief to reflect that the coin may be biased.

The analytical difficulty in this calculation lies in computing $P(D) = \int P(D|\theta)P(\theta)\mathrm{d}\theta$, which is often near impossible for realistically complex models. Fortunately modern computing power enables us to efficiently estimate our posterior distributions through algorithms such as Gibbs sampling and other Metropolis-Hastings schemes.

Hierarchical systems represent multi-variable models where some parameters depend on other parameters. Returning to the example of a coin flip, say the probability of heads (θ) is dependent on the factory in which the coin was minted. The probability that a coin was from a certain factory (ω) will then inform our value of (θ). Expressed mathematically, equation (1) now becomes:

$$\begin{aligned} P(\theta,\omega|D) &= \frac{P(D|\theta,\omega)P(\theta,\omega)}{P(D)} \\ &= \frac{P(D|\theta,\omega)P(\theta|\omega)P(\omega)}{P(D)}. \end{aligned} \tag{12}$$

This means that a prior distribution is only required for ω, as this distribution will directly inform our **conditional prior** of θ, via our model formulation. As such, when provided with data on coin flips from multiple coins from different factories, we obtain a posterior probability distribution of which factory a coin has come from, and the resulting probability of a coin flip resulting in heads. This structure of conditional independence means that data relating specifically to one parameter can still help inform the posterior of all other dependent variables, a key advantage of Bayesian inference.

A.2 Appendix 2 - Model Simulations of flock health

After confirming from model 5 the existence of variation in bird transition probabilities, we asked what the impact of this variation could be on the proliferation of *Campylobacter* STs. Using a previously published stochastic differential equation model of *Campylobacter* population dynamics within a broiler flock[19] we simulated two variant scenarios. Figure A1 displays a case study of the spread of five demographically identical strains of *Campylobacter* within a flock of 400 demographically identical broilers. Figure A1 shows that, as expected, all strains perform equally well and are equally represented in the amount being shed into the environment. Figure A2 instead shows the same model of five demographically identical strains of *Campylobacter* within a flock of 400 birds whose strength of immune response is drawn from a normal distributed centred around the value used for Figure 8. Figure A2E shows how five demographically equal strains can be sustained at broadly different levels across the flock due only to variation in bird immune response. This is caused by random chance, in that whichever strain is initially picked up by a super-shedder, such as the one shown in Figure A2D then sheds large amounts of that strain of *Campylobacter* into the environment, increasing the likelihood of then infecting other birds in the flock. This result greatly implies that the results shown in the data, whereby some STs seem to persist at higher levels than others in the flock, is likely due to the variation in bird transition probabilities, as opposed to phenotypic differences between STs.

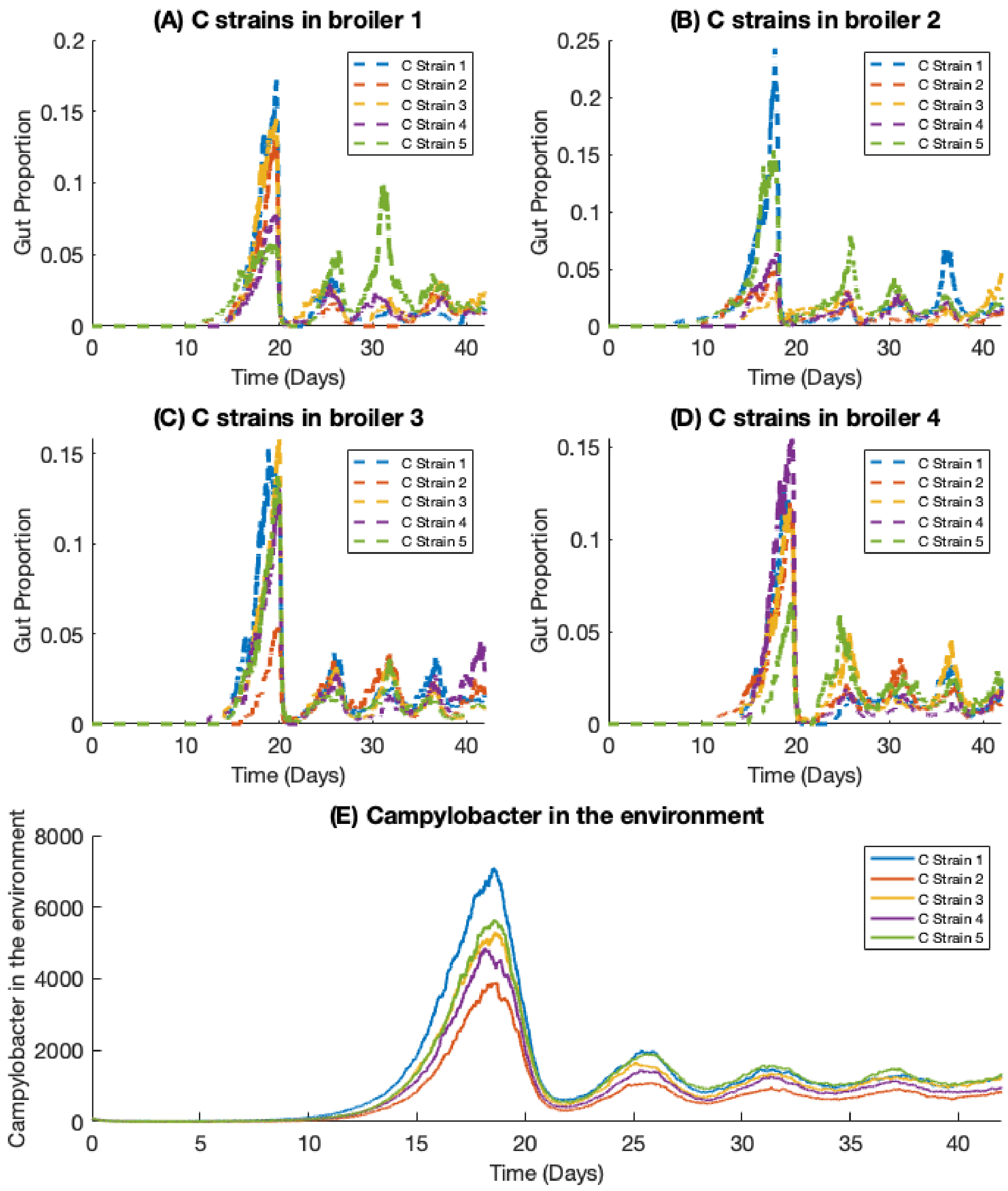

Figure A1. Dynamic behaviour of five identical strains of *Campylobacter* in a flock of identical broilers. (A) - (D) shows the population within the gut of individual broilers, while (E) displays the amount of *Campylobacter* in the environment, an expression of the average amount throughout the flock.

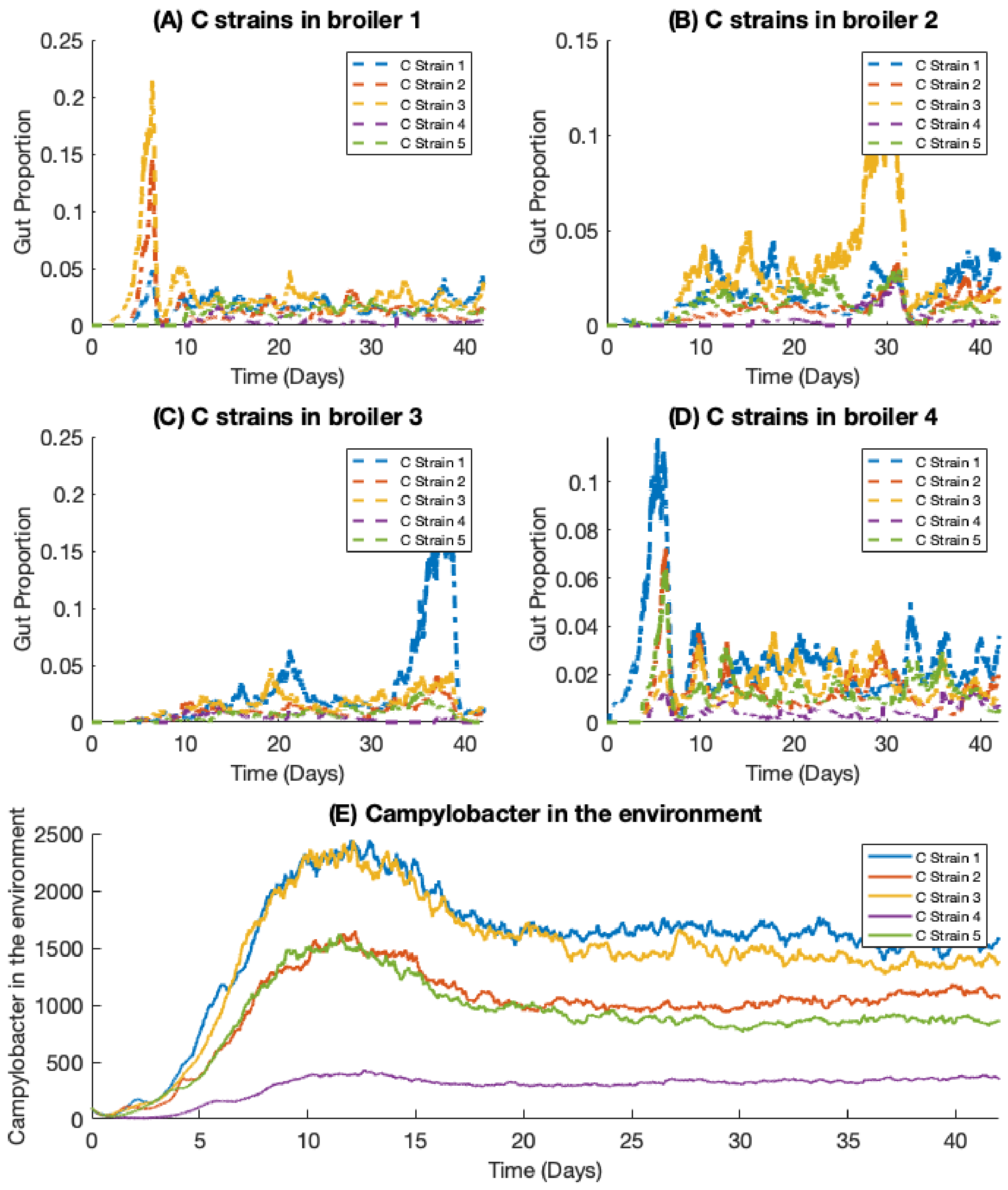

Figure A2. Dynamic behaviour of five identical strains of *Campylobacter* in a flock of broilers of varying susceptibility to infection. (A) - (D) shows the population within the gut of individual broilers, while (E) displays the amount of *Campylobacter* in the environment, an expression of the average amount throughout the flock.
